# Persistent autonomic dysfunction and bladder sensitivity in primary dysmenorrhea

**DOI:** 10.1038/s41598-019-38545-3

**Published:** 2019-02-18

**Authors:** Folabomi A. Oladosu, Kevin M. Hellman, Paula J. Ham, Laura E. Kochlefl, Avisek Datta, Ellen F. Garrison, Nicole D. Steiner, Genevieve E. Roth, Frank F. Tu

**Affiliations:** 1 Department of Obstetrics and Gynecology, NorthShore University HealthSystem, Evanston IL, 60201 USA; 2 Department of Obstetrics and Gynecology, Pritzker School of Medicine University of Chicago, Chicago IL, 60637 USA; 3 NorthShore Research Institute, NorthShore University HealthSystem, Evanston IL, 60201 USA

## Abstract

Menstrual pain, also known as dysmenorrhea, is a leading risk factor for bladder pain syndrome (BPS). A better understanding of the mechanisms that predispose dysmenorrheic women to BPS is needed to develop prophylactic strategies. Abnormal autonomic regulation, a key factor implicated in BPS and chronic pain, has not been adequately characterized in women with dysmenorrhea. Thus, we examined heart rate variability (HRV) in healthy (n = 34), dysmenorrheic (n = 103), and BPS participants (n = 23) in their luteal phase across a bladder-filling task. Both dysmenorrheic and BPS participants reported increased bladder pain sensitivity when compared to controls (*p’s* < 0.001). Similarly, dysmenorrheic and BPS participants had increased heart rate (*p’s* < 0.01), increased diastolic blood pressure (*p’s* < 0.01), and reduced HRV (*p’s* < 0.05) when compared to controls. Dysmenorrheic participants also exhibited little change in heart rate between maximum bladder capacity and after micturition when compared to controls (*p* = 0.013). Our findings demonstrate menstrual pain’s association with abnormal autonomic activity and bladder sensitivity, even two weeks after menses. Our findings of autonomic dysfunction in both early episodic and chronic visceral pain states points to an urgent need to elucidate the development of such imbalance, perhaps beginning in adolescence.

## Introduction

Menstrual cramping pain, clinically known as dysmenorrhea, often leads to several days of acute suffering every month throughout a woman’s reproductive years. Over 50% of reproductive-age sufferers report reduced quality of life due to disabling pain^1,2^. Primary dysmenorrhea, defined as menstrual pain without any anatomical contributing factors, is also a risk factor for developing other chronic pelvic pain conditions such as bladder pain syndrome (BPS). BPS, frequently comorbid with dysmenorrhea^3–5^, is a chronic visceral pain disorder defined by reported pain and heightened irritative symptoms (urgency, frequency) perceived to arise in the bladder and surrounding pelvic region^6–8^. Although the mechanisms underlying BPS are not completely understood, rodent models have shown that uterine inflammation can lead to cross organ sensitization of the bladder^9^ and urethra^10^, a process in part involving autonomic afferents^11^. Endometriosis, a potential cause of dysmenorrhea, has also been shown in a rat model to cause bladder inflammation and reduced micturition thresholds^12^. Identifying the processes that predispose dysmenorrheic women to BPS is imperative because current treatments for BPS are often ineffective^7^. Although secondary dysmenorrhea (also a risk factor for BPS) can be often addressed surgically^13^, primary dysmenorrhea is often responsive to over-the-counter medications^14^, making it a risk factor that is also therapeutically easy to target. Uncovering the mechanisms that link acute menstrual pain to chronic bladder pain will assist in creating strategies that mitigate bladder pain development.

A possible mechanism contributing to the development of chronic pelvic pain within dysmenorrheic women may be autonomic dysregulation. Autonomic cardiac activity, measured by heart rate variability (HRV), reflects homeostatic processes that appropriately respond to environmental stimuli or stressors. However, HRV is pathologically reduced by early life adverse events^15,16^ and chronic work stress^17^. Repeated exposure to such stressors could disturb normal autonomic function^15,16^, causing either persistent, inappropriately increased sympathetic activity or reduced parasympathetic activity. Reduced parasympathetic activity is highly associated with many chronic pain disorders^18^, including BPS^19–21^. Furthermore, reduced HRV is linked to impaired descending inhibition of spinal afferent neural activity in women with chronic pain^22^. If repeated episodic exposure to painful menses can fundamentally alter autonomic balance, it may also be a vulnerability pathway to developing a chronic pelvic pain state. In order to appropriately examine this hypothesis, however, we first need to examine autonomic balance within dysmenorrheic women across the menstrual cycle.

Dysmenorrheic women exhibit reduced parasympathetic activity during their menses^23–25^. It is unknown, however, if this abnormal autonomic activity is transient or persists across the menstrual cycle. The persistence of reduced parasympathetic activity during non-menses would suggest that autonomic dysfunction is not only acutely present during menses but sustained in dysmenorrheic women throughout the menstrual cycle. Therefore, we recorded HRV during the luteal phase to test the hypothesis that HRV is persistently reduced in women with dysmenorrhea and BPS compared to control participants.

Additionally, to specifically investigate the role of autonomic dysregulation in bladder sensitivity, we examined HRV profiles during natural bladder filling. We hypothesized that dysmenorrheic women may exhibit altered HRV profiles due to attentional bias during bladder filling and subsequent emptying. We have observed potential attentional bias related effects in a prior cross-sectional questionnaire based study^26^. Because psychological disorders such as depression and anxiety are associated with dysmenorrhea^27^ and dysfunctional autonomic activity^28^, we also explored whether psychological factors underlie associations between dysmenorrhea and autonomic activity.

## Results

### Demographics

Table 1 presents the demographic characteristics of the participants in this study. There were significant differences in race, age, and body mass index (BMI) across the cohorts (*p’s* < 0.05). Enrolled women with dysmenorrhea were more likely to be African-American (23.1%) than healthy controls (5.9%). BPS participants were more likely to be White-American (78%) than women with dysmenorrhea (51.0%) or healthy controls (55.9%). Women with dysmenorrhea were of comparable age and BMI to healthy controls. In contrast, BPS participants were six years older than healthy controls or women with dysmenorrhea and had about 10% higher BMI (*p’s* < 0.05).

**Table 1:** Demographic characteristics and blood pressure of all participants.

	Healthy Controls (n = 34)	Groups		P-value (Compared to Healthy Controls)	
		Dysmenorrhea (n = 103)	BPS (n = 23)		
	Mean (SEM)	Mean (SEM)	Mean (SEM)	Dysmenorrhea	BPS
Age	23.7 (1.1)	23.9 (0.6)	**30.0 (1.2)**	0.860	**<0.001**
BMI	22.3 (0.3)	23.2 (0.5)	**25.2 (1.3)**	0.366	**0.023**
**Blood Pressure**					
Systolic (mmHg)	110.2 (1.7)	112.7 (0.9)	113.9 (2.3)	0.200	0.168
Diastolic (mmHg)	68.6 (1.3)	**73.2 (0.8)**	**75.1 (1.7)**	**0.005**	**0.004**
	**N (%)**	**N (%)**	**N (%)**	Dysmenorrhea	BPS
**Race**				**0.025**	**0.016**
White	19 (55.9%)	**53 (51.0%)**	**18 (78.3%)**		
African American	2 (5.9%)	**24 (23.1%)**	**4 (17.4%)**		
Asian American	11 (32.4%)	**15 (14.4%)**	**0 (0.0%)**		
Other	2 (5.9%)	**12 (11.5%)**	**1 (6.7%)**		
**Education**				0.081	0.204
Completed HS	2 (5.9%)	9 (8.7%)	3 (13.0%)		
Some College	18 (52.9%)	48 (46.1%)	7 (30.4%)		
Associate’s	1 (2.9%)	4 (3.8%)	4 (17.4%)		
Bachelor’s	3 (8.8%)	29 (27.9%)	3 (13.0%)		
Post Graduate	10 (29.4%)	14 (13.5%)	6 (26.1%)		

Bold indicates mean values with standard error of the mean (SEM) significantly different than healthy controls (*p* < 0.05).

### Clinical Characteristics

Most of the dysmenorrheic participants had the primary form (97%). Three participants reported a history of endometriosis and one participant was discovered to have small subserosal and intramural leiomyomata (<2.5 × 2.5 cm) with ultrasonography. As expected, in healthy controls, menstrual pain was minimal (18 ± 3 mm, 0–100 mm VAS) and never led to missed school or work (Table 2). In contrast, women with dysmenorrhea reported severe menstrual pain (73 ± 2 mm), and missed school or work due to menstrual pain 2.0 ± 0.2 days over three months. Women with dysmenorrhea also reported more bladder symptoms (9.7 ± 0.8, GUPI) than healthy controls (3.2 ± 0.5, *p* < 0.001). Despite differences in bladder and menstrual pain, there were no differences in anxiety (*p* = 0.35) or depression (*p* = 0.48) measures between women with dysmenorrhea and healthy controls. Women with BPS reported severe bladder symptoms (30.2 ± 1.4, GUPI) and menstrual pain (75 ± 5 mm), frequently missed school or work due to pain (8.8 ± 3.2 days/3 months) and had higher levels of anxiety (*p* = 0.01) and depression (*p* = 0.018).

**Table 2:** Self-report measures of menstrual pain, bladder pain, and psychological characteristics of all participants.

	Groups			P-value (Compared to Healthy Controls)	
	Healthy Controls (n = 34)	Dysmenorrhea (n = 103)	BPS (n = 23)		
	Mean (SEM)	Mean (SEM)	Mean (SEM)	Dysmenorrhea	BPS
Absenteeism- Menstrual Pain (Days/3 months)	0.0 (0.00)	**2.0 (0.2)**	**8.80 (3.2)**	**<0.001**	**<0.001**
Menstrual Pain (0–100 VAS)	18.1 (3.1)	**73.3 (1.5)**	**74.5 (5.0)**	**<0.001**	**<0.001**
GUPI	3.2 (0.5)	**9.7 (0.8)**	**30.2 (1.4)**	**<0.001**	**<0.001**
PROMIS Anxiety	53.6 (1.2)	55.0 (0.8)	**59.3 (1.4)**	0.348	**0.009**
PROMIS Depression	50.5 (1.5)	51.7 (0.8)	**56.1 (2.0)**	0.483	**0.018**

Bold indicates mean values with standard error of the mean (SEM) significantly different than healthy controls (*p* < 0.05).

### Differences in baseline autonomic activity in participants with dysmenorrhea and BPS compared to healthy controls

**Figure 1:**
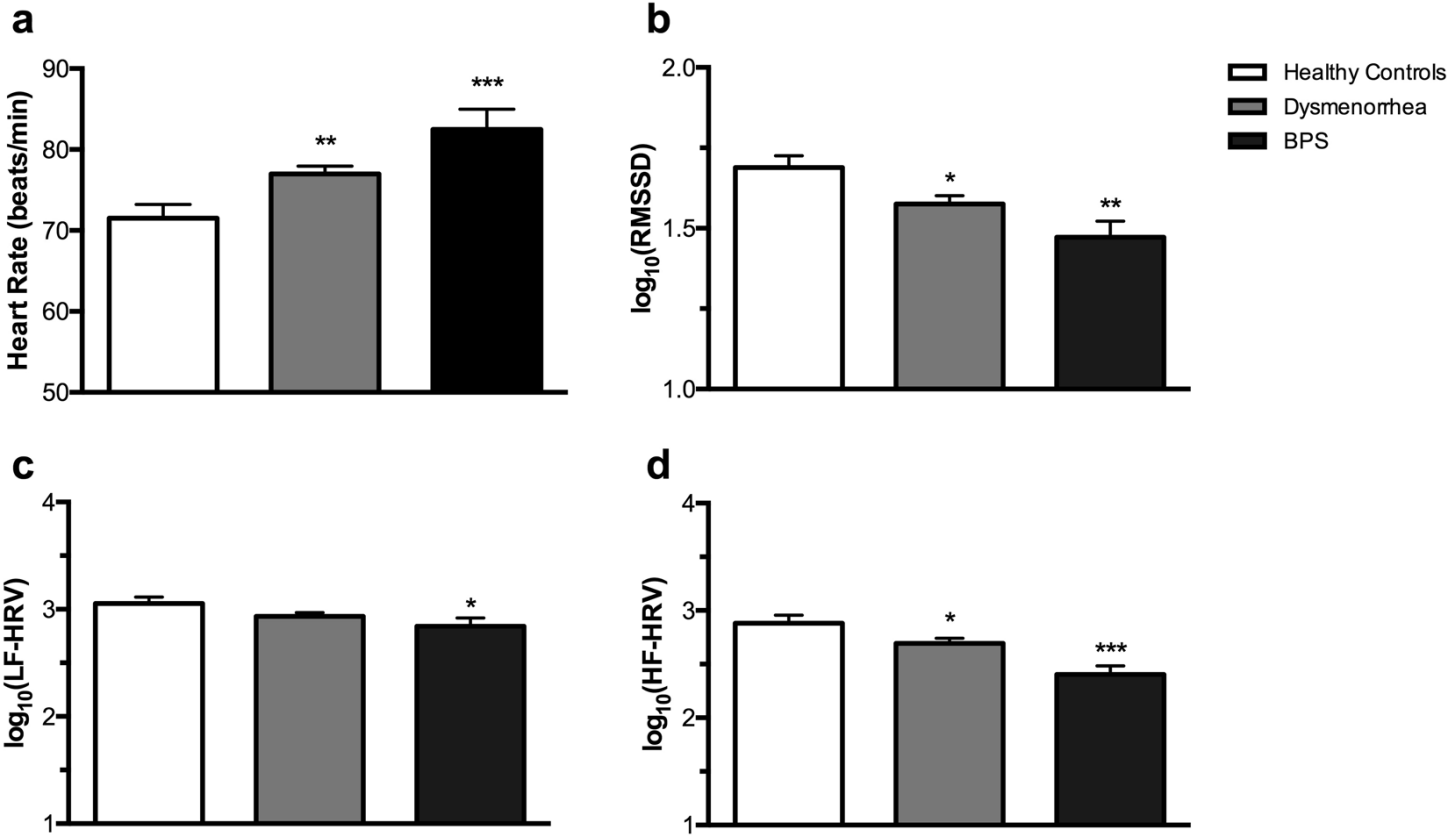
The three cohorts have distinct baseline HRV characteristics. (**a**) Heart rate, (**b**) RMSSD, (**c**) LF-HRV, and (**d**) HF-HRV were collected during rest prior to water ingestion. Data are represented as mean ± SEM. Data related to panels B–D were log_10_ transformed for normal distribution. *p < 0.05, **p < 0.01, and ***p < 0.005.

Baseline assessments across the three groups revealed distinct autonomic profiles (Table 1, Fig. 1). While systolic blood pressure was similar between the three groups (Table 1: *p’s* ≥ 0.168), dysmenorrheic and BPS participants had significantly higher diastolic blood pressure (73.2 ± 0.8 mmHg, *p* = 0.005; 75.1 ± 1.7 mmHg, *p* = 0.004, respectively) when compared to healthy controls (68.4 ± 1.3 mmHg). Dysmenorrheic and BPS participants also exhibited increased resting heart rate (76.9 ± 0.9 beats/min, *p* = 0.007 and 82.5 ± 2.5 beats/min, *p* < 0.001, respectively) compared to healthy controls (Fig. 1a; 71.5 ± 1.7 beats/min). In addition to heart rate and blood pressure, we evaluated three metrics of HRV: the root mean square of successive R-R interval differences (RMSSD), the spectral measures of high frequency power (HF-HRV) and low frequency power (LF-HRV). RMSSD was significantly reduced in dysmenorrheic and BPS participants (Fig. 1b: 1.57 ± 0.03, *p* = 0.019 and 1.47 ± 0.05, *p* = 0.002, respectively) compared to healthy controls (1.69 ± 0.04). As expected, similar effects were observed for HF-HRV (Fig. 1c). Interestingly, BPS participants had lower LF-HRV (Fig. 1d: 2.84 ± 0.08, *p* = 0.033) when compared to control participants.

### The Effect of Natural Bladder Filling on HRV

**Figure 2:**
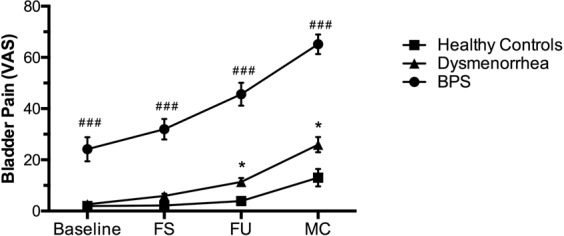
The three cohorts have different bladder pain sensitivities during bladder filling. Participants reported bladder pain sensitivity at baseline and at first sensation (FS), first urge (FU), and maximum capacity (MC) during bladder filling. Data are represented as mean ± SEM. *p < 0.05, **p < 0.01, and ***p < 0.005 (Dysmenorrhea vs Healthy Controls), ^#^*p* < 0.05, ^##^*p* < 0.01, ^###^*p* < 0.005 (BPS vs Healthy Controls).

**Figure 3:**
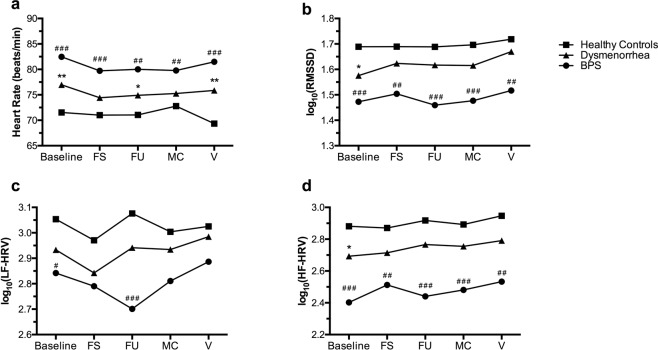
The effects of bladder filling on HRV characteristics. (**a**) Bladder filling did not significantly impact heart rate across the three cohorts expect following micturition (or void; **V)**. When compared to MC, healthy controls at V display a decrease in heart rate; this drop in heart rate is not observed in dysmenorrheic or BPS participants. (**b**,**d**) Bladder filling did not significantly impact RMSSD or HF-HRV across all three cohorts. (**c**) Bladder filling had a dynamic effect on LF-HRV in all groups. Both healthy controls and dysmenorrhea participants display a similar pattern during the bladder fill. In contrast, BPS participants have a lower and distinct LF-HRV pattern during bladder fill. Data are represented as mean ± SEM. Data related to panels B–D were log_10_ transformed for normal distribution. *p < 0.05, **p < 0.01, and ***p < 0.005 (Dysmenorrhea vs Healthy Controls). ^#^*p* < 0.05, ^##^*p* < 0.01, ^###^*p* < 0.005 (BPS vs Healthy Controls).

Next, we examined the effect of natural bladder filling on reported pain and HRV. As we have previously reported^29^, dysmenorrheic participants reported more pain during bladder filling than healthy controls (Fig. 2, *p* < 0.001). BPS participants reported more extensive bladder pain sensitivity than dysmenorrheic participants and healthy controls. During the bladder fill, group differences remained significant for heart rate, RMSSD, and HF-HRV (*p’s* < 0.001). Nevertheless, there was no overall significant effect of time during bladder filling on heart rate (*p* = 0.73), RMSSD (*p* = 0.58), or HF-HRV (*p* = 0.25) across the groups (Fig. 3a,b,d). However, there was an effect of time on LF-HRV (*p*λ0.04) across all groups. Both healthy controls and dysmenorrheic participants exhibit a similar pattern of changes in LF-HRV during the bladder fill task. In contrast, BPS participants exhibited a distinct and reduced LF-HRV pattern (*p *< 0.001), notably at first urge (Fig. 3c). Given their reduced bladder volumes^30^, BPS participants reached first urge more rapidly (36 ± 4 min) than dysmenorrheic participants (48 ± 1 min, *p* = 0.023). The difference in time to reach first urge with dysmenorrhea compared to healthy controls was minimal (55 ± 4 min, *p* = 0.094).

**Figure 4:**
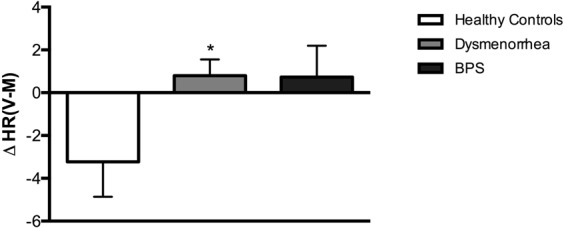
Dysmenorrheic and BPS participants fail to show post-void drop in heart rate. The difference in heart rate between after voiding and maximum capacity was calculated [ΔHR(V-M)] for all participants. Unlike healthy controls, dysmenorrheic and BPS participants did not display a drop in heart rate following micturition. Data are represented as mean ± SEM. *p < 0.05, **p < 0.01, and ***p < 0.005.

Divergent group effects were also observed following micturition (Figs 3a and 4). Dysmenorrheic participants exhibited little change in heart rate between maximum capacity and following micturition when compared to healthy controls (Fig. 4, *p* = 0.013). Heart rate changes in BPS participants were similar in magnitude to dysmenorrheic participants, but due to its reduced sample size, statistical significance was marginal when compared to healthy controls (Fig. 4, *p* = 0.082*)*.

### Factors underlying elevated heart rate in dysmenorrhea

We performed a *post-hoc* analysis within the dysmenorrheic and healthy control participants to evaluate the hypothesized biological and psychological mechanisms that could underlie differences in heart rate (Table 3). Baseline heart rate was significantly correlated with average prior self-reported menstrual pain (r = 0.23, *p* = 0.008) and with age (r = −0.26, *p* = 0.003). Notably, heart rate is lower in older adults, but we observed heart rate to be the highest within the older BPS cohort. Effects on heart rate were also unrelated to a participant’s race (African American: *p* = 0.918; Asian American: *p* = 0.28). We confirmed demographic factors do not confound any of the primary outcomes differences reported in this study with an ANOVA including race and age as covariates (Table 4). There was no significant correlation between baseline heart rate and bladder pain sensitivity (r = 0.16, *p* = 0.069), depression (r = −0.01, *p* = 0.915), or anxiety (r = 0.07, *p* = 0.427). Thus, even though participants were at least two weeks removed from menstruation, self-reported prior menstrual pain severity was the dominant factor associated with elevated heart rate.

**Table 3:** Pearson correlation coefficients between baseline heart rate and menstrual pain, bladder pain, age, and race (dysmenorrhea participants and healthy controls only).

	Heart Rate	P-value
Menstrual Pain	**0.233**	**0.008**
Bladder Pain	0.160	0.069
Age	**−0.262**	**0.003**
Anxiety	0.07	0.427
Depression	−0.01	0.915
African American	−0.009	0.918
Asian American	−0.095	0.280

Bold indicates significant coefficients (*p* < 0.05).

**Table 4:** Group differences between HRV variables are not affected by race or age.

HRV Variables	Unadjusted Group Differences (P-value)	Dysmenorrhea vs HC, Controlling for Race (P-value)	BPS vs HC, Controlling for Age (P-value)
Systolic BP	0.317	0.188	0.375
Diastolic BP	**0.006**	**0.005**	**0.016**
Heart Rate	**0.001**	**0.005**	**0.001**
LF-HRV	0.084	0.125	0.125
RMSSD	**0.005**	**0.014**	**0.006**
HF-HRV	**0.001**	**0.025**	**0.001**

P-values for group differences and for posthoc ANCOVA comparisons controlling for race and age, with regards to dysmenorrheic and BPS participants respectively, are shown. The effect of group differences on each factor was analyzed with an unadjusted one-way ANOVA. P-values are also shown for posthoc ANCOVA to evaluate the significant differences between dysmenorrhea vs healthy controls controlling for African American status. P-values are also shown for posthoc ANCOVA to evaluate the significant differences between BPS vs healthy controls controlling for age. Significantly different factors are bolded (*p* < 0.05). There was not a significant effect of African American status or age on primary outcome variables.

## Discussion

In the presented study, distinct bladder pain sensitivity and autonomic characteristics differentiated the three cohorts. Participants with dysmenorrhea and BPS exhibited higher bladder pain sensitivity, elevated heart rate and diastolic blood pressure, and reduced cardiac parasympathetic activity (i.e. RMSSD and HF-HRV) when compared to healthy controls. Divergent heart rate effects between groups were also observed after micturition. Menstrual pain severity—but not bladder pain sensitivity, depression, or anxiety—was the primary factor associated with elevated heart rate.

Unlike our study, prior studies on HRV in dysmenorrhea have focused on the menstrual phase. During the menstrual phase, Wang and colleagues observed women with dysmenorrhea (n = 66) had lower LF-HRV and HF-HRV during menstruation when compared to healthy controls (n = 54)^25^. However, a smaller study reported that women with dysmenorrhea (n = 10) had increased HF-HRV during the first two days of menstruation and increased LF-HRV (measured from systolic blood pressure) during the last three days of menstruation^23^. Thus, our study reflects reduced parasympathetic activity observed in women with dysmenorrhea in the larger study likely extends to the luteal phase.

Notably, our results with BPS are also similar to other published studies^19,21^. Given that dysmenorrhea (which most often starts at menarche) most often occurs before BPS onset (average age of first symptoms: 30 to 47)^31^, decreased parasympathetic activity reported in BPS likely occurred prior to diagnosis due to comorbid dysmenorrhea. It is unclear, given that this is a cross-sectional study, if repeated bouts of severe menstrual pain promote altered autonomic function or if women with reduced parasympathetic tone have a predisposition to develop dysmenorrhea and other chronic pelvic pain disorders. Future longitudinal studies investigating HRV in adolescents with menstrual pain will be key to define the contributions from autonomic dysregulation.

If autonomic dysregulation is a key factor in the development of chronic pain, it is critical to introduce appropriate preventive treatments that enhance parasympathetic tone. One such candidate treatment, HRV biofeedback, maximizes respiratory sinus arrhythmia and HF-HRV to increase parasympathetic activity^32^. When used in children with functional abdominal pain and IBS, 2 months of HRV biofeedback treatment increased HF-HRV, reduced pain intensity and pain frequency^33^. A smaller follow-up study demonstrated that two weeks of HRV biofeedback was associated with an absence of clinical symptoms in 63% of participants^34^. Pilot studies of HRV biofeedback for other chronic pain conditions are promising^35–37^, but their therapeutic effectiveness must be assessed within BPS patients and dysmenorrheic women.

The only significant effect of natural bladder filling on autonomic parameters was LF-HRV. Reduced LF-HRV in BPS at first urge could be due to the short time these patients take to reach this cystometric threshold. Nevertheless, patients with BPS had lower LF-HRV even at baseline. Other researchers have reported that patients with BPS have reduced HRV^19,21,38,39^, but have not specifically studied LF-HRV. Although we observed group and time differences in LF-HRV during bladder filling, we cannot draw firm conclusions about the implication of these findings due to the ambiguous definition of LF-HRV. Initially thought to be a measure of sympathetic activity^40^, LF-HRV is considered to reflect baroreflex sensitivity independent of sympathetic activity when participants are at rest^41–43^. Based on this definition of LF-HRV, our findings suggest that BPS participants have reduced baroreflex sensitivity, which would indicate improper inhibition of sympathetic activation and improper parasympathetic activation. Other researchers posit, however, that LF-HRV is neither a measure of sympathetic activity nor baroreflex sensitivity^44^. Further research is needed to clarify the significance of LF-HRV and to establish its significance in chronic pain conditions such as BPS.

Another intriguing finding from this study is the lack of decreased heart rate in dysmenorrheic and BPS participants following micturition. Fagius and Karhuvaara showed that sympathetic activity steadily increased during natural bladder filling and decreased following void in healthy men^45^. We also observed this phenomenon in healthy controls, but not in dysmenorrheic or BPS participants, potentially a result of altered sympathetic activity. However, given that the present study did not directly measure sympathetic activity, we cannot definitively state that sympathetic tone is altered in visceral pain states. Thus, future research must investigate sympathetic activity within these two populations with more explicit measures.

Depression and anxiety, frequently comorbid with chronic pain disorders, are also associated with increased heart rate^46,47^. In our study, however, we did not find a significant correlation between these psychological factors and resting heart rate. This observation is most likely due to the fact dysmenorrheic participants are most often not clinically depressed or anxious^26,48^. On a related note, our cross-sectional study of dysmenorrhea has similarly shown a correlation between non-menstrual pelvic pain and menstrual pain independent of anxiety or depression^26,48^. Thus, menstrual pain is associated with changes in autonomic function and pain risk independent of psychological factors.

Advantages of this study include: (1) examination of the effects of dysmenorrhea on HRV during the luteal phase; (2) use of natural bladder filling, which allowed for the examination of the effect of bladder filling without comprising the integrity of HRV measurements; (3) verification of primary dysmenorrhea diagnoses by comprehensive questionnaires and clinical exam; and (4) validated, standardized psychometric questionnaires. Limitations to this study include the absence of comparative bladder pain sensitivity and HRV data during menses. Since continuous measurement of blood pressure or nerve activity was not obtained, it was not possible to directly evaluate sympathetic activity amongst participants.

In conclusion, our study demonstrates that dysmenorrheic women present elevated heart rate, reduced parasympathetic tone, and impaired micturition-mediated reflex during the luteal phase. The abnormal autonomic profile in dysmenorrheic women is similar to those of BPS participants; yet clinical onset typically predates their diagnosis by a decade. Although the identification of the key factors responsible for pain chronification requires further study, episodic menstrual pain is a stimulus sufficiently associated with alterations in autonomic activity and bladder sensitivity, persisting into the luteal phase. Our identification of autonomic abnormalities, both in young women with episodic visceral pain and in slightly older women with chronic visceral pain, points to the need for longitudinal studies characterizing how autonomic dysfunction relates to pain states.

## Methods

### Participant Recruitment

This cross-sectional study was designed to characterize uterine cross-organ influences on bladder pain. This study was performed in accordance with Health Insurance Portability and Accountability Act privacy guidelines and was approved by the NorthShore University HealthSystem Institutional Review Board. Written informed consent was obtained before participation. Female participants, ages 18–45, were recruited with flyers posted in the community (including local college campuses and businesses), through contact via the Illinois Women’s Health Registry, and by referral from gynecology clinics in our health system. Participants included in this study were recruited and enrolled from August 2014 to November 2017. Potential participants were instructed to contact our team and complete a phone screen. At the phone screen, we initially confirmed participants met eligibility criteria (see detailed inclusion/exclusion criteria below). Eligible participants were required to between the ages of 18–45, have regular menses, BMI < 40, be willing to abstain from oral contraceptive pills, and not have more than 5 migraines with aura in the past year (a potential contraindication for oral contraceptive pills).

Eligible participants were scheduled for an initial screening visit. This is a substudy from a larger prospective study to investigate the mechanisms of sensory hypersensitivity in women with visceral pain disorders. The larger study focused heavily on women with dysmenorrhea, and used standard quantitative sensory tests such as pressure pain algometry, visceral distension, and conditioned pain modulation, while also assessing for other postulated contributors to pain sensitivity, including autonomic measures, psychosocial profiles, and endocrine measurements^29^. The parent study design heavily skews the sample size for this substudy towards women with dysmenorrhea by a ratio of 4 to 1 to enrich for phenotypic characterization.

### Screening Visit

Participants completed questionnaires encompassing medical, surgical, psychological, and gynecological history. All data was entered into REDCAP with automatic field range restriction^49^. A gynecologist or research nurse who is trained in pelvic pain evaluation and blinded to participant identity performed a standardized pelvic exam on all participants. Consistent with standard practice in our tertiary gynecology referral clinic, this includes systematic palpation of the perineum, vagina, pelvic floor, fornixes, uterus, cervix, urethra and bladder with explicit capture of areas of tenderness, presence of any uterovaginal prolapse, and presence of any palpable pelvic masses such as ovarian cysts, or leiomyoma^50^. A simplified version of the sonographic bladder test (detailed below under Assessment Visit), referred to as the *rapid bladder test*, was conducted at the screening visit in participants with dysmenorrhea. They were asked to drink 20 ounces (591 mL) of water within 5 minutes and report when they felt 1] first sensation (when they first felt capable of urinating) and 2] first urge (when they would ordinarily void). At each of these times, participants recorded their bladder urgency and bladder pain on 0–100 Visual Analog Scale (**VAS**; 0: no pain/urgency, 100: worse pain/urgency imaginable).

All participants were provided luteinizing hormone urinary assay kits (Wondfo USA Co., Willowbrook, IL) to time participation for the subsequent mid-luteal phase assessment visit (approximately 17–25 days post-onset of menses). The severity of their menstrual and bladder pain was rated using web-based daily diaries throughout their menstrual cycle on a 0–10 numerical rating scale (**NRS**; 0: No pain, 10: Worst pain imaginable) preceding the assessment visit.

### Inclusion and Exclusion Criteria

To be enrolled as a healthy control, a participant was required to rate menses pain ≤ 3 (0–10, NRS) and to have no concurrent chronic pain diagnoses. Enrolled dysmenorrheic participants were required to rate menses pain ≥ 4 (0–10, NRS) and have no concurrent chronic pain diagnoses. Enrolled BPS, along with having either primary or secondary dysmenorrhea, participants were required to have a prior diagnosis of BPS as defined by American Urological Association^7^ for more than three months with average pelvic pain ≥ 3 (0–10, NRS) and pressure or discomfort related to the bladder accompanied by at least one other urinary symptom such as persistent urge to void or frequency.

Participants were excluded from the assessment visit for the presence of active pelvic or abdominal malignancies, the absence of regular menses, active genitourinary infection in the last four weeks, the inability to read or comprehend the informed consent in English, the refusal to undergo pelvic examination/testing, hypertension, and the refusal to withdraw from oral contraceptives for two months prior to the study visit.

Of the initial 288 participants screened, 79 participants were excluded because they were not qualified to continue in the study based on criteria (n = 46), they decided not to continue (n = 14), or because they were lost to follow up (nλ19). Of the remaining 209 screened participants who qualified for the assessment visit, 16 were excluded because they were classified as chronic pain or chronic pelvic pain participants, 4 had ventricular arrhythmia, and 29 had inadequate or unavailable EKG signals.

### Assessment Visit

Prior to this visit, participants were asked to avoid taking short-acting, over-the-counter analgesics (i.e., ibuprofen, acetaminophen), opioids, and caffeine for at least six hours. All visits were scheduled to take place during the participant’s luteal phase.

The non-invasive bladder test, a mimic of clinical retrograde cystometry, was performed on all participants^51^. All participants emptied their bladder prior to the test. Participants were asked to drink 20 ounces (591 mL) of water within 5 minutes and report when they reached three standard cystometric urgency thresholds: first sensation, first urge, and maximum capacity^52^. At baseline, micturition, and at each of these times, the bladder volume was quantified with three-dimensional sonographic measurements (GE Voluson 750, Wauwatosa, WI), and participants rated their bladder pain and urgency on a 0–100 VAS^51^. Participants were asked to ingest an additional 10 ounces (296 mL) of water at 45 minutes and 60 minutes. To limit burden, participants were given 120 minutes following water consumption to rate bladder pain and urgency. If a participant reached the time limit, she was instructed to report her pain and urgency at 120 minutes regardless of reaching maximum capacity. Only seven participants failed to reach maximum capacity within the allotted time; only their reported first sensation and first urge thresholds were included in this manuscript.

While waiting for their bladder to fill, participants completed the questionnaires. The NIH Patient Reported Outcomes Measurement Information System (PROMIS) anxiety and depression scales^53^ were administered to evaluate psychological profiles, while we characterized relative bladder pain syndrome symptomatology using the Genitourinary Pain Index (GUPI)^54^.

### Heart Rate Variability Measures and Analyses

To collect heart rate variability data during bladder filling, standard EKG electrodes (3M Company, St. Paul, MN) were placed on the upper left and right pectoralis muscles (just below the clavicle) and on the participant’s left side near the base of the ribcage. EKG activity was sampled at a rate of 1 kHz and amplified by 1000 with an AC BIOPAC amplifier (Biopac Systems, Aero Camino Goleta, CA) and filtered between 1–250 Hz to isolate heart rate activity.

Collected raw EKG data were processed using AcqKnowledge Software (Biopac Systems, Aero Camino Goleta, CA). Activity was analyzed according to guidelines set by the Task Force of the European Society of Cardiology and the North American Society of Pacing and Electrophysiology^55^ Reviewers blinded to participant identity selected an artifact free 4-minute segment at baseline. A smaller segment (2 minutes) was used to avoid artifacts and select activity at the three cystometric thresholds, and 2 a minute-segment following micturition. Selected data segments were bandpass filtered (low frequency cutoff: 5 Hz, high frequency cutoff: 35 Hz). After being filtered, data segments were screened for artifacts (i.e. missed heartbeat, participant movement, and R-R intervals < 0.5 s or > 1.5 s). Segments containing less than five artifacts were appropriately edited to remove these while those containing more than five entirely omitted. Segments were also omitted if there was evidence of non-sinus rhythm or inadequate (i.e. too short) EKG signals.

After being cleaned, segments are analyzed using the open-source HRVAS package in MATLAB (MathWorks Inc, Natick, MA). Heart rate, systolic blood pressure and diastolic blood pressure were measured to determine overall degree of activation at rest and during natural bladder filling (heart rate only). Additionally, we measured time and frequency domain variables associated with HRV. The primary dependent variable in the time domain was the root mean square of successive R-R interval differences (RMSSD), which reflects the beat-to-beat variance in heart rate and is typically used to estimate parasympathetic changes in heart rate variability^56^. The primary dependent variables for the frequency domain were the spectral measures of low frequency power (LF-HRV) and high frequency power (HF-HRV). LF-HRV encompasses a power spectrum that ranges from 0.04–0.15 Hz; what it reflects is still up for debate (see discussion). HF-HRV ranges from 0.15–0.40 Hz, reflects parasympathetic activity and is highly correlated to RMSSD^56^. The log_10_ was applied to RMMSD, LF-HRV, and HF-HRV to adjust normality of the distribution.

### Statistical Analyses

All analyses were performed in SAS version 9.3. Complete data sets were obtained for all analyzed participants. Outliers, individual time points with HRV exceeding 1.5 times the interquartile range, were excluded according to Tukey’s rule^57^.

Our sample sizes were unequal because the parent study focused largely on dysmenorrhea and enrolled participants were used for analyses. To confirm our ability to identify a difference in autonomic factors between the groups, a sensitivity power analysis α = 0.05, 1-β = 0.8) was performed. We had adequate power for detecting a medium effect size (d = 0.56) difference between healthy controls and dysmenorrheics and a large effect size (d = 0.78) difference healthy controls and BPS participants. To accommodate potential effects of unequal sample sizes, we confirmed the homogeneity of variance in all analyses. After confirmation of normality, two-tailed Student’s T-test with *p* < 0.05 threshold for significance was used to determine group differences in demographic characteristics (Table 1), clinical characteristics (Table 2), baseline HRV assessments (Fig. 1), and heart rate differences between maximum capacity and void (Fig. 3). A two-way ANOVA was used to examine an effect of group and time (i.e., natural bladder filling) on bladder pain sensitivity and HRV variables (Fig. 2). Pearson correlation coefficients were calculated to examine possible associations between baseline heart rate and bladder pain sensitivity, menstrual pain sensitivity, age, body mass index, being African-American, and being Asian-American (Table 3). The ANOVA p-values for the primary outcome differences and post-hoc group differences accounting for demographics as a covariate were also calculated (Table 4).

## Data Availability

The datasets generated and analyzed during the current study are available from the corresponding author upon request.
